# Histopathological patterns of bovine tuberculosis in cattle slaughtered in Southern Brazil: correlation with molecular detection of *Mycobacterium* spp

**DOI:** 10.1007/s11259-026-11347-5

**Published:** 2026-06-12

**Authors:** Taina dos Santos Alberti, Kauê Rodriguez Martins, Virgilio Zoppi Lemos, Clairton Marcolongo-Pereira, Marta Santos de Moraes, Margarida Buss Raffi, Rodrigo Casquero Cunha, Eliza Simone Viégas Sallis

**Affiliations:** 1 Laboratório Regional de Diagnóstico (LRD), Faculdade de Veterinária, Universidade Federal de Pelotas (UFPel), Campus Capão do Leão s/n, Capão do Leão, RS 96015-560 Brazil; 2 Programa de Pós-Graduação em Patologia, Faculdade de Medicina de Botucatu, Universidade Estadual Paulista “Júlio de Mesquita Filho” (UNESP), Campus Botucatu, Av. Prof. Mário Rubens Guimarães Montenegro, s/n, Botucatu, SP 18618687 Brazil; 3 Programa de Pós-Graduação em Ciências da Saúde, Centro Universitário do Espírito Santo (UNESC), Av. Fioravante Rossi 2930, Bairro Martinelli, Colatina, 29703-858 ES Brazil

**Keywords:** Bovine tuberculosis, *Mycobacterium* spp., Granuloma, Histopathology, PCR, Slaughterhouse surveillance

## Abstract

Bovine tuberculosis (bTB) is a chronic infectious disease caused by *Mycobacterium bovis*, which has major veterinary, economic, and zoonotic relevance. This study aimed to characterize the histopathological patterns of bTB lesions and their association with molecular detection in cattle slaughtered in Southern Brazil. Thirty-six tissue samples with lesions suggestive of bTB were collected during municipal inspection in Pelotas, Rio Grande do Sul. Polymerase chain reaction (PCR) targeting the 16 S rRNA gene was used to detect *Mycobacterium* species. DNA in 35/36 samples (97.2%), whereas Ziehl–Neelsen (ZN) staining revealed acid-fast bacilli in 23/36 cases (61.1%), a statistically significant difference (McNemar’s exact test, *p* = 0.0018). Grossly, the lesions ranged from caseous nodules to purulent and cavitary forms. Histologically, two main morphological patterns were identified: classical granulomatous lesions with complete fibrous encapsulation (19/36, 52.8%) and disorganized granulomatous inflammation lacking complete encapsulation (17/36, 47.2%). Multinucleated giant cells were present in all ZN-positive cases, whereas neutrophilic infiltration was significantly more frequent in disorganized lesions than in classical granulomas (Fisher’s exact test, *p* = 0.0002), suggesting active inflammation and impaired granuloma containment. PCR showed higher sensitivity than ZN staining, detecting mycobacterial DNA even in lesions with a low bacillary load. As the assay targeted the 16 S rRNA gene, results were reported as *Mycobacterium* spp., and species-specific methods would be required for the definitive identification of *M. bovis*. These findings reinforce the diagnostic value of combining histopathology and molecular methods and emphasize the importance of recognizing atypical lesion patterns for the accurate detection of bovine tuberculosis in slaughterhouse surveillance systems.

## Introduction

Tuberculosis (TB) is a chronic infectious disease caused by members of the *Mycobacterium tuberculosis* complex (MTC). In cattle, it is primarily caused by *Mycobacterium bovis*, a highly pathogenic bacterium with significant zoonotic potential (Palmer et al. 2019; Dalto and Pavarini 2023; Larenas-Muñoz et al. 2024; Alberti et al. 2025). Bovine tuberculosis (bTB) remains a significant concern for animal and public health worldwide and is responsible for economic losses due to carcass and organ condemnation in slaughterhouses (Palmer et al. 2007).

Bovine tuberculosis is characterized by progressive granulomatous lesions affecting multiple organs, particularly the lymph nodes, lungs, and liver (Dalto and Pavarini 2023; Terefe 2014; Alberti et al. 2020). Gross lesions typically present as yellowish-white nodules with caseous and mineralized areas, often surrounded by a fibrous capsule and occasionally coalescing into extensive necrotic areas (Palmer et al. 2019; Dalto and Pavarini 2023).

Microscopically, lesions consist of granulomatous inflammation with central caseous necrosis, frequently associated with dystrophic mineralization, surrounded by epithelioid macrophages, multinucleated giant cells, and lymphocytes, and are commonly delimited by fibrous tissue (Palmer et al. 2019). Although Ziehl–Neelsen staining is widely used to detect acid-fast bacilli, its sensitivity may be limited in lesions with a low bacillary load, emphasizing the importance of complementary diagnostic techniques, such as molecular methods (Dalto and Pavarini 2023).

Several studies have evaluated diagnostic approaches for bTB in slaughtered cattle, highlighting the limitations of each method when used independently. Furlanetto et al. (2012) demonstrated that complementary methods, including histopathology and molecular techniques, improved the detection of tuberculous lesions during post-mortem inspection. Similarly, Dametto et al. (2020) reported that the association of multiple diagnostic analyses increased the accuracy of confirming bTB in dairy cattle. Silva et al. (2018) compared PCR and ZN staining in slaughtered bovines and showed that molecular methods presented greater sensitivity, particularly in samples with a low bacillary load or advanced tissue changes. Despite these findings, studies specifically addressing the correlation between histopathological patterns and molecular detection of *Mycobacterium* spp. in naturally infected cattle remain scarce, underscoring the need for further investigation in slaughterhouse surveillance settings.

In this context, the present study aimed to characterize the molecular and histopathological patterns of lesions associated with bTB in southern Brazil and to confirm the infection using polymerase chain reaction (PCR).

## Materials and methods

Bovine fresh tissue samples (lymph nodes, lungs, and liver) with lesions suggestive of bTB were submitted to the Regional Diagnostic Laboratory, Faculty of Veterinary Medicine, Federal University of Pelotas (LRD/FV/UFPel), Brazil. Samples were collected from cattle slaughtered under municipal inspection between March 2023 and February 2024.

Upon arrival, the samples were examined macroscopically and classified as homogeneous, granular, purulent or cavitary. Lesion distribution was categorized as focal, focally extensive, multifocal, multifocal, coalescing, or diffuse.

Representative tissue fragments were fixed in 10% buffered formalin for 48 h, routinely processed, embedded in paraffin, sectioned at 3 μm, and stained with hematoxylin and eosin (HE) and Ziehl–Neelsen (ZN) stains.

Histopathological evaluation was performed using a semi-quantitative scoring system, grading the lesions as mild (+), moderate (++), or marked (+++), according to the intensity and extent of the histological changes, with analyses performed independently by three pathologists (Gibson-Corley et al. 2013). The following parameters were assessed: well-structured granulomas, caseous necrosis, fibrosis, dystrophic mineralization, and predominant inflammatory cell types (macrophages, epithelioid cells, multinucleated giant cells, lymphocytes, and neutrophils).

Granulomas were classified as well-organized when they presented with central caseous necrosis, with or without mineralization, surrounded by mononuclear inflammatory cells, and delimited by a fibrous capsule. Lesions lacking complete encapsulation or presenting disorganized inflammatory infiltrates were classified as having disorganized granulomatous inflammation.

Multinucleated giant cells were quantified per high-power field and graded as mild (+; 1–2 cells), moderate (++; 3–5 cells), or marked (+++; >6 cells). The remaining inflammatory cell populations were evaluated semiquantitatively. All histological evaluations were performed independently by three pathologists, and discrepancies were resolved by consensus agreement.

For molecular analysis, representative tissue fragments were refrigerated and submitted to the Veterinary Molecular Biology Laboratory (LaBMol-Vet), Faculty of Veterinary Medicine, UFPel, Brazil. DNA extraction was performed using the Brazol reagent (LCG Biotecnologia, Brazil) following the manufacturer’s instructions. DNA concentration and purity were assessed spectrophotometrically, and the samples were standardized to 50 ng/µL.

PCR targeting the 16 S rRNA gene of *Mycobacterium* spp. was performed using primers MYCGEN-F and MYCGEN-R (Wilton and Cousins 1992), generating a 1030 bp amplicon. Each 25 µL reaction contained 2× PCR MasterMix (Ludwig Biotecnologia, Brazil), 250 nM of each primer, 2 µL template DNA, and nuclease-free water to complete the final PCR volume. All samples were tested in triplicate, including the positive and negative controls.

Amplification was carried out with an initial denaturation step at 95 °C for 3 min, followed by 35 cycles at 92 °C for 30 s, 62 °C for 30 s, and 72 °C for 60 s, with a final extension step at 72 °C for 5 min. Amplicons were resolved by agarose gel 1.5% electrophoresis and visualized under ultraviolet (UV) light.

Statistical analyses were performed using Fisher’s exact test to assess the association between histopathological variables and ZN staining results, and McNemar’s exact test was used to compare the sensitivity of PCR and ZN staining. Statistical significance was set at *P* < 0.05.

Samples were obtained during routine post-mortem inspection, with no experimental procedures performed on the animals; therefore, ethical approval was not required.

## Results

During the study period, 36 samples presenting gross lesions suggestive of bovine tuberculosis were obtained from cattle slaughtered in seven slaughterhouses under municipal inspection in the municipality of Pelotas, Rio Grande do Sul, Brazil, with each sample corresponding to a different animal. In 31 cases, only lymph nodes were received; in four cases, lung tissue and lymph nodes were received; and in one case, liver tissue and lymph nodes were received. The lymph nodes evaluated included the mediastinal, tracheobronchial, retropharyngeal, apical, and hepatic lymph nodes, with the first three being the most frequently affected.

**Fig. 1:**
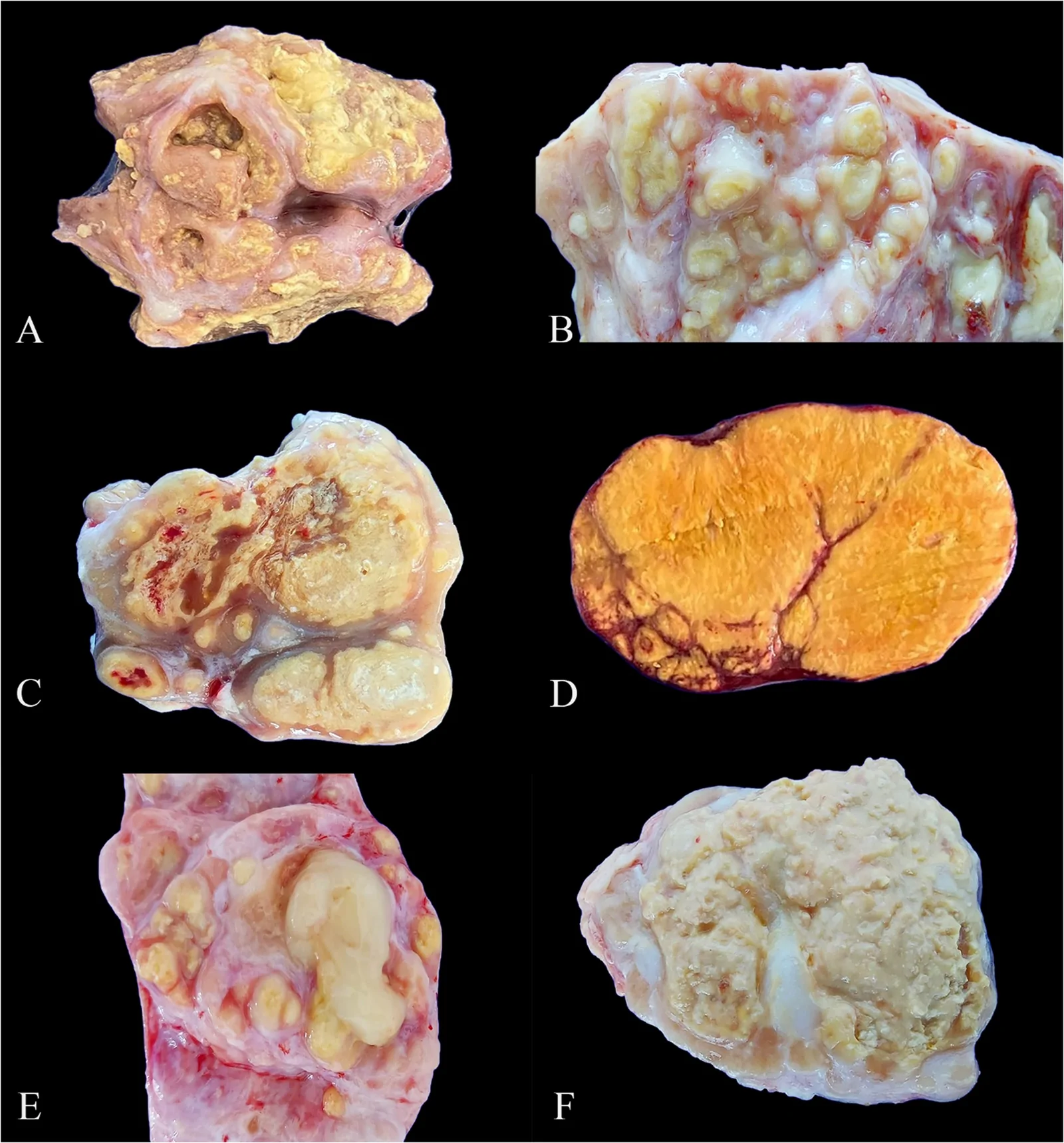
Gross lesions observed in the lymph nodes of cattle with tuberculosis. **A**: Lymph node with a focal, well-demarcated area containing yellowish granular material with a caseous appearance. **B**: Lymph nodes exhibiting small, yellowish, firm, granular nodules that are multifocal to coalescing. **C**: Lymph node with multifocal to coalescing, yellowish, firm areas and regions of gelatinous material. **D**: Lymph node surface entirely replaced by a homogeneous, yellowish, firm, and granular lesion. **E**: Lymph node exhibiting focal areas of purulent material containing viscous yellow material. **F**: Lymph node exhibiting a focally extensive area of yellowish, granular material with a pasty consistency

Macroscopically, most lesions consisted of yellowish nodules with firm, granular, and caseous cut surfaces, distributed in focal (Fig. 1A), multifocal to coalescing (Fig. 1B and C), or diffuse patterns (Fig. 1D). Purulent areas containing viscous yellow material (Fig. 1E) and cavitary lesions with pasty and granular contents (Fig. 1F) were also observed.

In two cases, the lymph nodes showed marked enlargement, irregular cut surfaces, and loss of corticomedullary architecture. Histologically, these cases exhibited lymphoid depletion associated with epithelioid macrophage infiltration. PCR confirmed the presence of *Mycobacterium* spp., whereas ZN staining was negative in both cases.

**Fig. 2:**
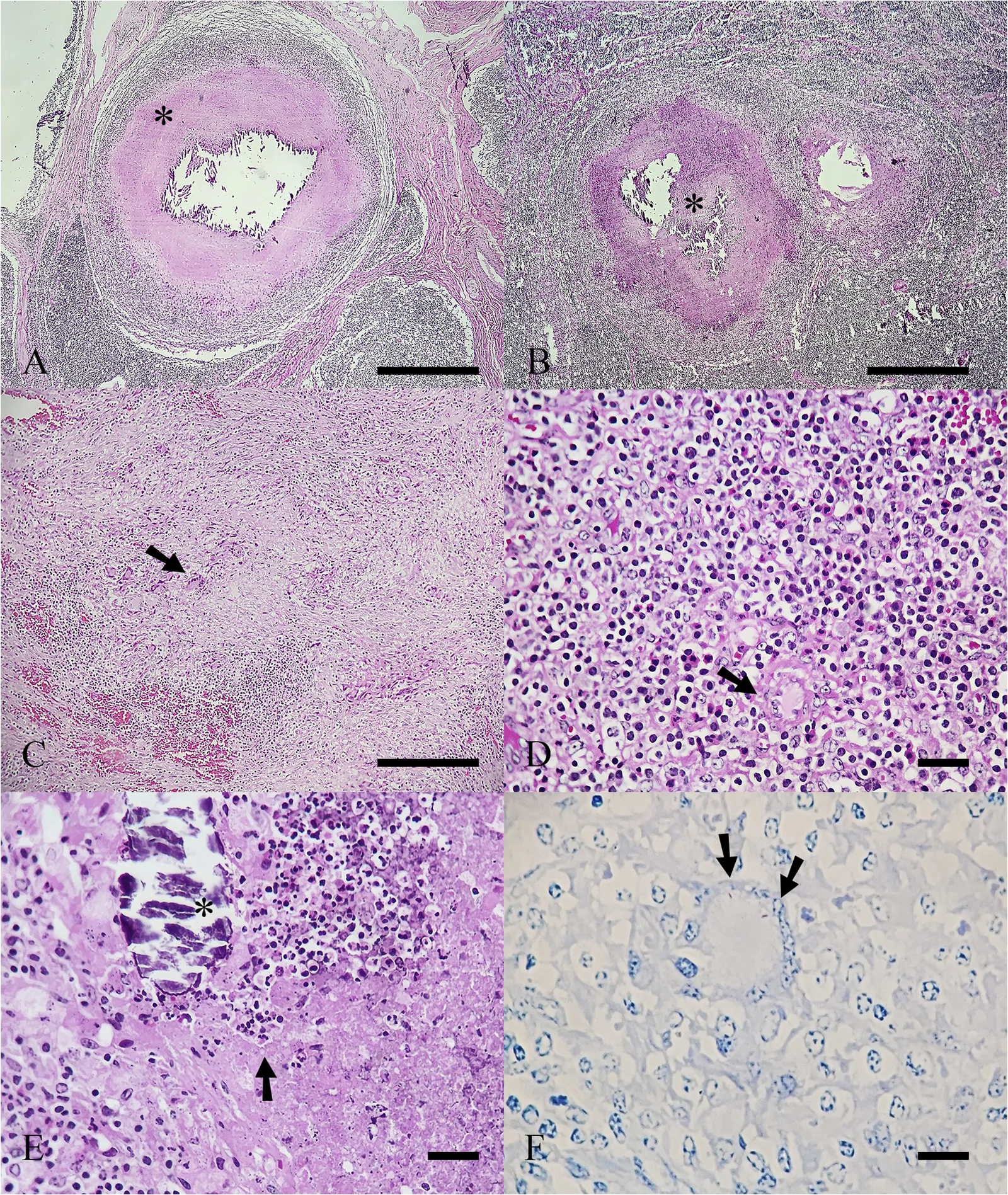
Histopathological lesions in bovine tuberculosis in lymph nodes. **A**: Well-organized granuloma characterized by central caseous necrosis (*) and dystrophic mineralization, surrounded by a mononuclear inflammatory infiltrate and delimited by a fibrous capsule (classical granulomatous pattern). HE. Scale bar = 100 μm. **B**: Disorganized granuloma characterized by central necrosis (*) without fibrous encapsulation and a mononuclear inflammatory infiltrate composed of lymphocytes and macrophages. HE. Scale bar = 100 μm. **C**: Disorganized granulomatous lesion with Langhans-type multinucleated giant cells (arrows) and a mixed inflammatory infiltrate composed of lymphocytes and macrophages. HE. Scale bar = 100 μm. **D**: Disorganized granulomatous lesion with Langhans-type multinucleated giant cells (arrows) and mixed inflammatory infiltrate. HE. Scale bar = 20 μm. **E**: Area of dystrophic mineralization (*) associated with neutrophilic inflammatory infiltrate (arrow). HE. Obj. 20×. **F**: ZN staining showing two acid-fast bacilli (arrows) within a Langhans-type multinucleated giant cell. ZN. Scale bar = 20 μm

Histopathological evaluation identified well-organized granulomas in of 19/36 samples (52.8%), characterized by central caseous necrosis, dystrophic mineralization, and fibrous encapsulation (Fig. 2A). The remaining 17/36 samples (47.2%) exhibited disorganized granulomatous inflammation, lacking complete fibrous encapsulation (Fig. 2B and C). Based on these findings, two main morphological patterns were identified: (1) classical granulomatous lesions (52.8%, 19/36) and (2) disorganized granulomatous lesions (47.2%, 17/36).

Multinucleated giant cells were present in all ZN-positive lesions (Fig. 2D e F) and varied in quantity from mild (+) in 6/22 cases to moderate (++) in 3/22 cases and marked (+++) in 13/22 cases. Macrophages, epithelioid cells, and lymphocytes were consistently observed with moderate to high intensity across all samples.

Neutrophils (Fig. 2E) were detected in 27/36 samples (75%) and were significantly more frequent in disorganized lesions (15/17; 88.2%) than in classical granulomas (5/19; 26.3%) (Fisher’s exact test, *p* = 0.0002). Dystrophic mineralization (Fig. 2E) was observed in 33/36 samples (91.7%), with marked mineralization more frequent in well-organized granulomas. Fibrosis was present in 34/36 samples (94.4%), either forming a well-demarcated capsule or appearing irregularly distributed throughout the inflammatory infiltrate without complete encapsulation.

No significant association was observed between granuloma organization and ZN positivity (Fisher’s exact test, *p* = 1.00). Neutrophilic infiltration was observed in both ZN-positive and ZN-negative lesions. No significant association was observed between ZN positivity and dystrophic mineralization (Fisher’s exact test, *p* = 0.539) or fibrosis (*p* = 0.124).

PCR detected *Mycobacterium spp.* DNA in 35/36 samples (97.2%), whereas ZN staining revealed acid-fast bacilli (AFB) in 23/36 cases (61.1%) (Table 1). Among the PCR-positive samples, 22/35 (62.9%) were ZN-positive. One sample was ZN-positive but PCR-negative, whereas 13 samples were PCR-positive and ZN-negative. Considering PCR as the reference method, the sensitivity of ZN staining was 62.9% (Table 1).

**Table 1:** Comparison of PCR and Ziehl–Neelsen staining results for detection of *Mycobacterium* spp. in bovine tissue samples

Diagnostic test	Positive	Negative	Total
PCR	35	1	36
Ziehl–Neelsen	23	13	36

## Discussion

The present study demonstrated the occurrence of *Mycobacterium* spp. infection in cattle slaughtered under municipal inspection in Pelotas, Rio Grande do Sul, highlighting bovine tuberculosis as a persistent public health concern and reinforcing its importance as a major cause of granulomatous lesions in this region (Alberti et al. 2020).

It is important to note that the PCR assay used in this study targeted the 16 S rRNA gene, which is a highly conserved genetic marker among members of the genus Mycobacterium. Consequently, this approach does not allow species-level differentiation within the *Mycobacterium tuberculosis* complex (Sun et al. 2016), and positive samples are therefore reported as *Mycobacterium* spp. Definitive identification of *M. bovis* requires the use of species-specific molecular targets, such as IS6110, RD4 deletion typing, or spoligotyping-based methods (Jagielski et al. 2014; Tekle and Govena 2020). Accordingly, the inability to determine the infecting species is a limitation of the present study.

Comparison between PCR and ZN staining showed marked differences in their sensitivity. PCR detected mycobacterial DNA in 97.2% of the samples, while acid-fast bacilli in only 61.1% of the cases. McNemar’s exact test confirmed that this difference was statistically significant (*p* = 0.0018), reinforcing the greater sensitivity of molecular methods, especially in lesions with low bacillary loads, necrosis, autolysis, or prolonged fixation, as previously reported by Silva et al. (2018).

Although ZN staining remains an important histopathological tool, its sensitivity depends on the bacillary load and lesion preservation. In contrast to Dametto et al. (2020), who reported ZN positivity in all confirmed cases, only a proportion of samples were positive in the present study, likely reflecting differences in lesion stage and bacterial burden and highlighting the value of combining molecular and histopathological methods.

Macroscopically, most lesions showed the classical features of bovine tuberculosis, although purulent and cavitary lesions were also observed. Similar patterns have been observed in other infectious conditions, such as nonspecific lymphadenitis, actinobacillosis, and nocardiosis (Souza et al. 2016; Furlanetto et al. 2012; Tessele et al. 2014). These atypypical presentations likely reflect different stages of lesion evolution and reinforce the need to include bovine tuberculosis in the differential diagnosis during slaughter inspection.

In two PCR-positive cases, the typical macroscopic and histopathological features of tuberculosis were absent. This finding may be explained by the early stages of infection prior to granuloma formation and may also account for animals that are tuberculin-positive but lack visible lesions at post-mortem inspection. As suggested by Silva et al. (2018), the integration of molecular and histopathological methods increases diagnostic sensitivity and enables the detection of subclinical or early stage infections.

Histopathological evaluation identified two main morphological patterns: classical granulomatous lesions with complete fibrous encapsulation and disorganized granulomatous inflammation lacking complete encapsulation. The presence of classical granulomas in approximately half of the cases indicates chronic and well-established infections and corresponds to the typical tuberculous granuloma associated with type IV hypersensitivity responses (Palmer et al. 2022; Dalto and Pavarini 2023). The frequent observation of caseous necrosis, dystrophic mineralization, and fibrosis further supports the chronic nature of these lesions and highlights the morphological spectrum of bovine tuberculosis under natural conditions (Palmer et al. 2019; Dalto and Pavarini 2023).

Granuloma formation is closely associated with cytokine-mediated immune responses, particularly tumor necrosis factor (TNF) and interferon-gamma (IFN-γ), which are produced by activated macrophages and lymphocytes (Sasindran and Torrelles 2011). Experimental studies have shown that suppression of TNF leads to granuloma disorganization and reactivation of latent infection (Keane et al. 2001), whereas IFN-γ deficiency results in impaired granuloma formation and increased neutrophilic inflammation (Pearl et al. 2001). In nonhuman primates, TNF neutralization is associated with a disseminated disease (Lin et al. 2010). Although cytokine expression was not evaluated in the present study, the presence of disorganized lesions may reflect the variability in host immune responses.

Neutrophils were detected in 75% of the samples and were present in both organized and disorganized lesions, indicating the coexistence of acute and chronic inflammatory components. Experimental studies have shown that early lesions induced by *M. bovis* infection are initially characterized by neutrophilic infiltration, followed by the formation of granulomas (Palmer et al. 2019). Therefore, variable neutrophil infiltration likely reflects different stages of lesion progression or reactivation episodes.

Neutrophils are key components of the innate immune response and may contribute to the pathogenesis of chronic tuberculosis (Gaffney et al. 2022). In human tuberculosis, they are frequently associated with cavitary lesions and tissue destruction (Lyadova 2017). In the present study, neutrophilic infiltration was significantly more frequent in disorganized lesions (15/17; 88.2%) than in classical granulomas (5/19; 26.3%) (Fisher’s exact test, *p* = 0.0002), suggesting a possible association with active inflammation and impaired granuloma containment. This finding is consistent with experimental data showing that, at advanced stages of infection, large-scale neutrophilic infiltration is associated with exudative processes, lesion necrosis, and extracellular matrix degradation mediated by neutrophil-derived metalloproteinases, contributing to tissue destruction and lesion instability (Lyadova 2017; Gaffney et al. 2022).

Although Menin et al. (2013) associated neutrophils with increased bacterial load, the present study found that acid-fast bacilli were more frequently detected in lesions with marked multinucleated giant cells than in those with prominent neutrophilic infiltration. A marked number of giant cells were observed in most ZN-positive cases, suggesting their central role in mycobacterial sequestration within granulomatous lesions (Palmer et al. 2007).

The present study showed a high occurrence of *Mycobacterium* spp. infection in cattle slaughtered under municipal inspection in southern Brazil and highlighted the morphological heterogeneity of bovine tuberculosis lesions. Two main histopathological patterns were identified: classical and disorganized granulomatous lesions, reflecting different stages of lesion development and host immune responses. The frequent presence of neutrophils, even in well-organized granulomas, indicates the coexistence of acute and chronic inflammation.

The detection of acid-fast bacilli was more closely associated with multinucleated giant cells than with neutrophilic infiltration, suggesting a potential role for these cells in mycobacterial sequestration within granulomatous lesions. In addition, the higher sensitivity of PCR compared to that of ZN staining reinforces the importance of combining molecular and histopathological techniques for an accurate diagnosis, particularly in early or atypical lesions.

Overall, these findings expand our understanding of the morphological spectrum of bovine tuberculosis and support the use of integrated diagnostic approaches to improve detection in slaughterhouse surveillance systems.

## Data Availability

No datasets were generated or analysed during the current study.
